# Understanding public perspectives on genetically engineered Brinjal and the adoption of modern biotechnology in Bangladesh

**DOI:** 10.3389/fbioe.2024.1471201

**Published:** 2024-08-21

**Authors:** Sium Ahmed, Abdullah Mohammad Shohael, Tanvir Ahamed, Razu Ahmed, Shawon Ahmed, H. M. Shahid Hassan

**Affiliations:** ^1^ Cell Genetics and Plant Biotechnology Laboratory (CGPBL), Department of Biotechnology and Genetic Engineering, Jahangirnagar University, Dhaka, Bangladesh; ^2^ Department of Geography and Environment, Jahangirnagar University, Dhaka, Bangladesh

**Keywords:** Bt Brinjal, consumer attitude, modern biotechnology, public perception, science communication

## Abstract

The agricultural sector in Bangladesh is currently facing numerous challenges. The country is currently endeavoring to adopt modern biotechnological tools, such as genetic engineering, to modify crops with the aim of ensuring food security. Notably, Bt Brinjal represents a significant milestone as the first genetically engineered (GE) food crop commercially cultivated in South Asia. Public perception and awareness are crucial steps forward for accepting and commercializing GE crops within society. The study discussed here aims to assess public perception and awareness regarding modern biotechnology and GE crops, focusing mainly on Bt Brinjal in Bangladesh. A random survey considered demographic factors such as age, gender, hometown, educational qualification, and occupation to explore the public attitudes towards Bt Brinjal and modern biotechnology. Approximately one-third of those surveyed considered Bt Brinjal safe for consumption, and a third expressed a willingness to buy Bt Brinjal, while nearly two-thirds believed it would gain popularity in the market alongside other crops. Most respondents recognized the necessity of utilizing modern biotechnology for crop improvement beyond Bt Brinjal, and respondents with science backgrounds displayed higher awareness and a more positive attitude than those with limited education or non-science backgrounds. This study explores the public perceptions of Bt Brinjal and the adoption of modern biotechnology in Bangladesh by examining factors such as knowledge dissemination, acceptance levels, and concerns related to GE crops, and offers a meaningful perspective that can shape decision-making processes to promote agricultural sustainability and achieve relevant sustainable development goals in Bangladesh.

## Introduction

Bangladesh, situated in South Asia, has a rich agricultural landscape that has significantly contributed to its economy for centuries. Most people still rely heavily on agriculture as their primary source of income (Rahman, 2017). However, Bangladesh’s agricultural sector faces numerous challenges including salinity intrusions, pests, diseases, land fragmentation, water scarcity, and vulnerability to natural disasters like floods and cyclones frequently impeding crop productivity (M. H. Mondal, 2010). Modern biotechnology holds significant potential for transforming agricultural practices in Bangladesh. Advances in this field can help to overcome urgent difficulties by increasing nutrient efficiency, raising crop yield, and strengthening resistance to pests and diseases (Shohael and Hefferon, 2023).

To fully realize the potential of modern biotechnology in agriculture, it is imperative to understand and comply with the regulatory frameworks, biosafety concerns, and public acceptance while promoting inclusive and equitable access to biotechnological innovations among smallholder farmers (Shohael and Hefferon, 2023). While Bangladesh has made significant progress in developing a robust biosafety regulatory system to safely implement biotechnology advancements in agriculture (Khanam and Hasan, 2019), understanding public perspectives on GE crops and the broader adoption of modern biotechnology is essential for informed decision-making and sustainable agricultural progress (Siddiqui et al., 2022). Despite the significant implications of GE crop adoption for agriculture, food security, and environmental sustainability, there has been limited effort to engage the public in meaningful dialogue and understand their perceptions, concerns, and knowledge regarding GMOs. This lack of communication hampers the product’s performance in the market (Abdullah et al., 2018).

Bangladesh has been engaged in advanced crop biotechnology research since the late 1970s by applying plant tissue culture to different plant varieties (Choudhury and Islam, 2004). The application of genetic engineering in crop improvement started after 1990, while the formulation of biosafety regulations also started (Khanam and Hasan, 2019). Brinjal cultivars genetically engineered for insect resistance (Bt Brinjal) developed by the Bangladesh Agricultural Research Institute (BARI) with the support of the United States Agency for International Development (USAID) were given authorization for cultivation in Bangladesh by the National Committee on Biosafety on 30 October 2013 (A. M. Shelton et al., 2018).

Eggplant (*Solanum melongena L.*) is a popular plant species grown worldwide for its edible fruit with multi-dimensional use in cooking (Rotino, Sala, and Toppino, 2014). Popularly known as Brinjal in South Asia, it has become a part of the regular diet, a source of nutrition, and an essential source of income for many farmers (Frary, Doganlar, and Daunay, 2007). In Bangladesh, Brinjal, locally known as Begun, is a staple diet, and it ranks third after potato and rice in terms of consumption quantity, which makes it an essential component of food security (Ahsanuzzaman and Zilberman, 2018). However, the overall production of Brinjal is relatively low because of insect infestation, which damages the yield by two-thirds, despite efforts to introduce insecticide and other management practices (Ahsanuzzaman and Zilberman, 2018).

Since the approval, Bt Brinjal has been cultivated by the farmers and sold to consumers. The introduction of Bt Brinjal in Bangladesh marks a significant development for several reasons. It has encouraged more research and development using modern biotechnology, opening doors for creating more GE products, and presented farmers with the decision to adopt GE or non-GE crops by observing the benefits, while consumers can have their own choices between GE and non-GE crops by their quality.

Despite various studies examining the performance and benefits of Bt Brinjal, there has been a noticeable gap in understanding public perception towards this GE crop in Bangladesh. Little is known about public attitudes regarding Bt Brinjal and its background. The study discussed here explores the public perceptions of Bt Brinjal and the adoption of modern biotechnology in Bangladesh by examining factors such as knowledge dissemination, acceptance levels, and concerns related to GE crops. We aim to offer a meaningful perspective that can shape decision-making processes to promote agricultural sustainability and achieve relevant sustainable development goals in Bangladesh.

### A survey of public perceptions

**FIGURE 1:**
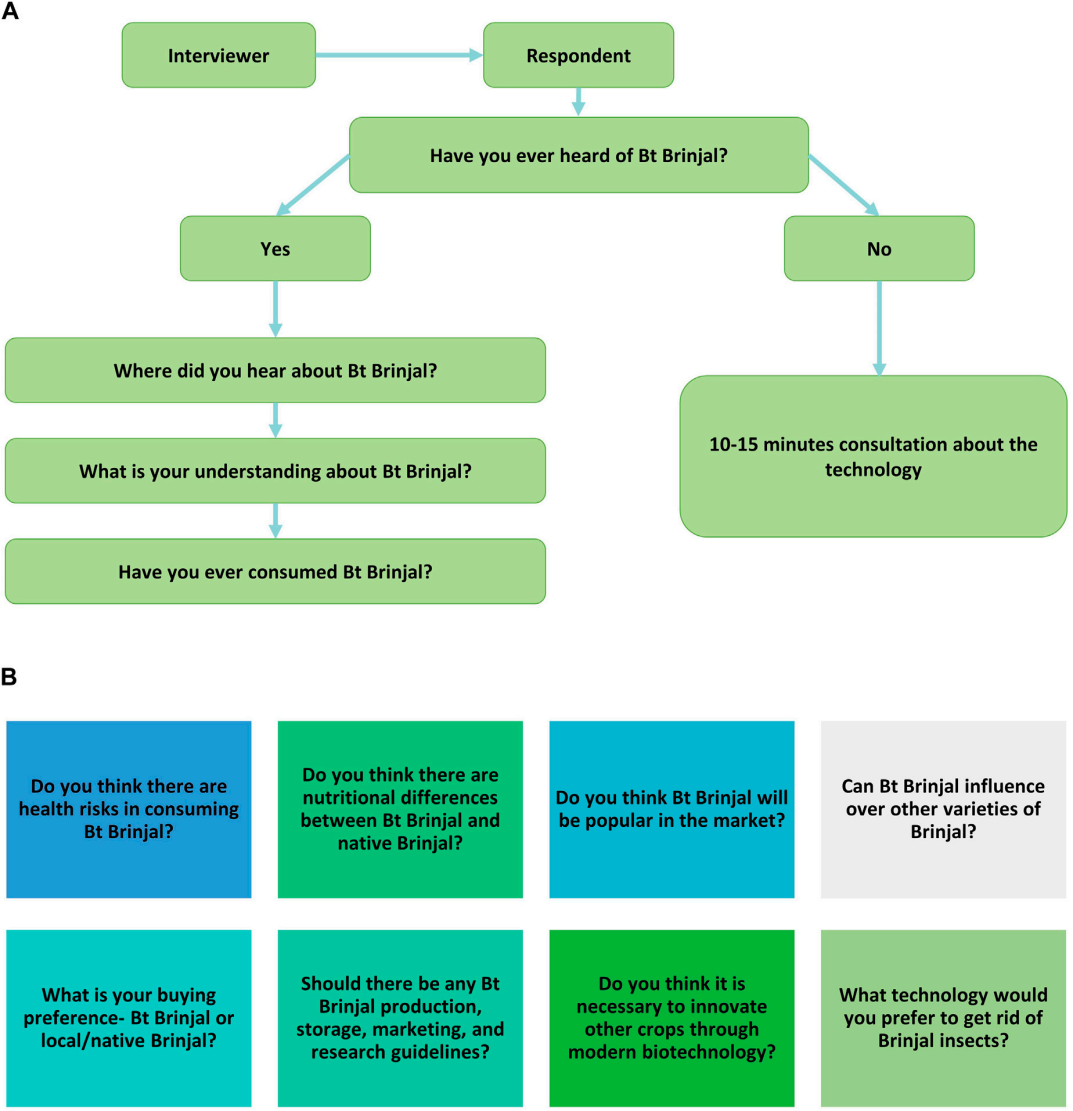
**(A)** The flow diagram of data collection; **(B)** The questions asked during the data collection process.

We prepared a questionnaire to gather insights from selected participants about their perceptions of Bt Brinjal and modern biotechnology. Questions were designed to obtain information on some socio-demographic variables and structured questions, including respondents’ knowledge of Bt Brinjal, understanding of the technology and its potential, consumption history, market impact, and opinions on the need for crop improvement through modern biotechnology (Figure 1). The survey was conducted randomly on 1000 willing participants, and their identities were kept anonymous. Ten data collectors conducted one-to-one interviews, each lasting approximately 20 min per individual.

### Demographics

A total of 1000 respondents participated in this study, comprising 637 males and 363 females. The age of the participants ranged from 18 to 75, with a mean age of 29.5 ± 11 years. Participants were sorted out based on their home districts. Bangladesh has 64 home districts. These districts encompass diverse ethnicities, cultures, agricultural practices, and food habits. The education level of participants varied, with 0.5% reporting to have a Ph.D., 13.0% reporting completion of postgraduate studies, 26.8% reporting completion of graduate degrees, 34.5% reporting completion of higher secondary certificate degrees, 7.8% reporting completion of secondary certificate degrees, 11.0% reporting completion of primary education and 6.4% were illiterate. Of 826 respondents who have studied above secondary studies, 39.9% had a science background, 23.2% had a business studies background, and 19.5% had a humanities background. Regarding employment status, 56.7% of respondents were students, 13.2% were in public or private service, 9.0% of participants were businessmen, 2.1% of participants were farmers, and 19.0% of participants were in other sectors of occupations (rickshaw pullers, labor, unemployed).

### Familiarity with Bt Brinjal, source of knowledge and idea about the trait

Among 1000 respondents, around 50.1% heard about Bt Brinjal, 44.4% did not, and 5.5% were unaware of the term. Of those who previously heard about Bt Brinjal, 70% had a science background in their study. 79% of respondents were students. 90.8% completed higher secondary education or above. Among the 501 respondents who were familiar with Bt Brinjal, 29.1% knew the term from coursework, 12.1% heard the term from mass media (Television, newspaper, etc.), 6.1% heard the term from their friends or relatives, and 2.8% knew from other sources. When asked about their knowledge of the trait, 22.9% of the respondents said that Bt Brinjal is insect-resistant, and 3.3% thought that Bt Brinjal is a high-yielding Brinjal variety. In comparison, 21.7% of the participants thought Bt Brinjal encompasses both insect resistance and high-yielding properties. Additionally, 2.2% of respondents thought Bt Brinjal has neither insect resistance nor high-yielding properties.

### Consumption, health risks and nutritional differences

Among the 501 respondents who were familiar with Bt Brinjal, about 15.5% respondents confirmed that they consumed Bt Brinjal at least once, 50.5% did not consume it, and 34.0% were unaware of whether they ever consumed Bt Brinjal or not. Among the respondents who already knew about Bt Brinjal, 24.1% thought there might be health risks, 52.1% thought there were no health risks associated with Bt Brinjal consumption, and 23.8% were uncertain. 73.3% thought there were nutritional differences between Bt Brinjal and local Brinjal, while 22.5% knew there were no nutritional differences, and 24.2% were uncertain. Among the 444 respondents who were consulted, 15.8% of respondents opined that Bt Brinjal may pose a health risk, while 21.4% thought there were no health risks, and 62.8% refused to pose any comment regarding the risk. 27.5% of the consulted respondents opined Bt Brinjal may have nutritional differences. In comparison, 14% of respondents opined Bt Brinjal is not nutritionally different from local varieties, and 58.6% refused to comment on Bt Brinjal’s nutritional composition.

### Preference in buying, popularity in the market, and positivity towards modern biotechnology

**FIGURE 2:**
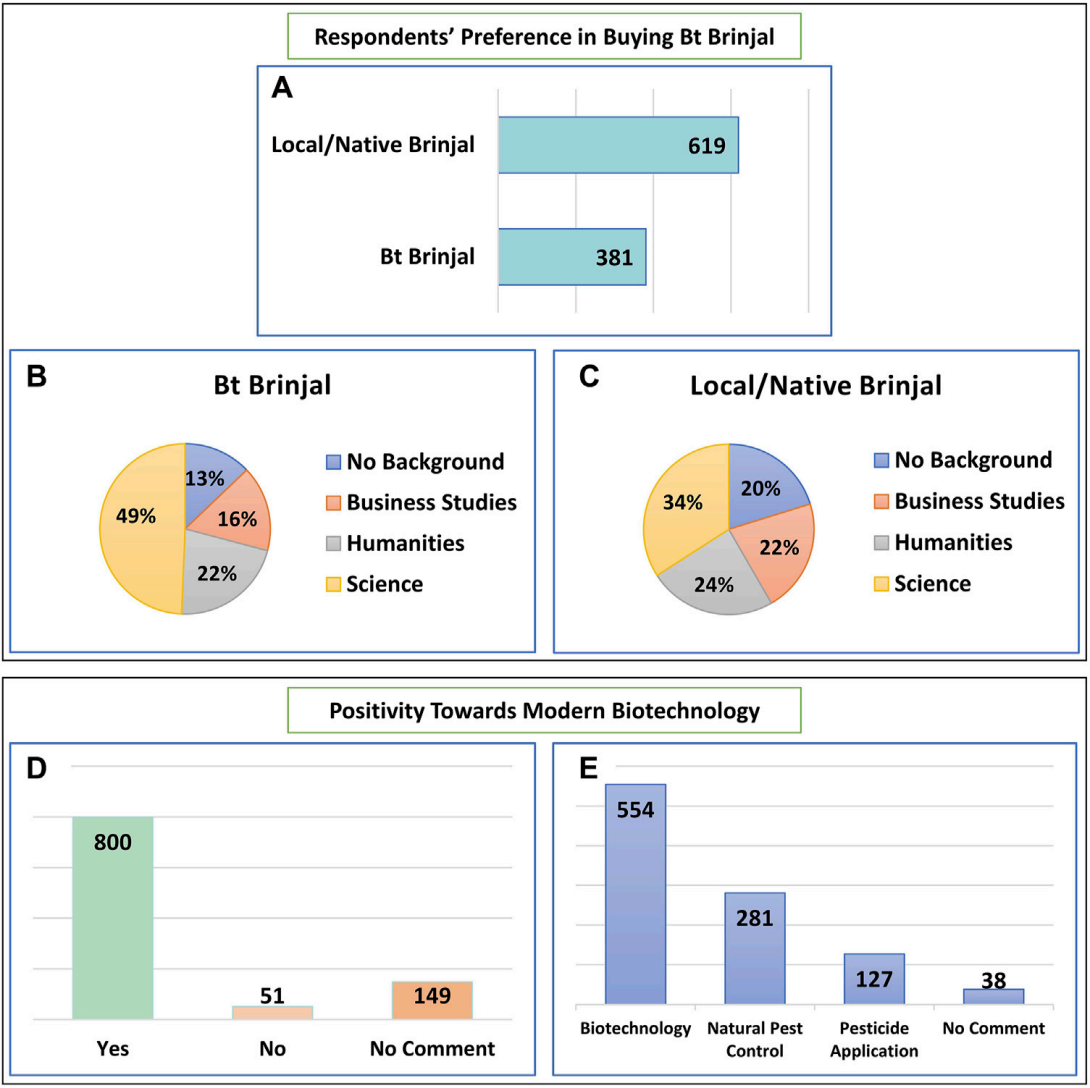
Respondents’ preference in buying Bt Brinjal and positivity towards modern biotechnology. **(A)** Number of respondents who would buy local/native Brinjal or Bt Brinjal while both are available in the market; **(B)** Educational background of the respondents who would prefer to buy Bt Brinjal; **(C)** Educational background of the respondents who would prefer to buy local/native Brinjal; **(D)** Respondent’s opinion regarding improvement of popular crops through modern biotechnology; **(E)** Respondent’s preference for controlling pests.

Figure 2 illustrates the respondent’s preference in buying Bt Brinjal and outlines the respondent’s positivity toward modern biotechnology. Among the respondents, more than 58.8% agreed that Bt Brinjal is likely to be popular in the market, 8.4% were not in agreement, and 32.8% did not provide any opinion. Of those who thought Bt Brinjal would be popular, 53% of the respondents were from a science background. 48.2% of respondents think that Bt Brinjal may influence and lead to the loss of popularity of other local/native varieties. 22.2% opposed this, and 29.6% were unwilling to comment on this. Among the respondents who thought Bt Brinjal might influence the loss of popularity of other varieties, 54% were from a science background.

Respondents were optimistic about the technology, but their preference skewed toward native/local Brinjal varieties. 38.1% of the respondents agreed they would buy Bt Brinjal if available in the market, while 61.9% think they preferred buying local Brinjal varieties. Of those who opined to buy Bt Brinjal, almost half (49%) were from a science background. On the other hand, of the participants whose preference was aligned with local Brinjal, 66% were from non-science backgrounds. While discussing the technology, the respondents were eager to see its benefits. If biotechnology is the answer, they would like to embrace it. Most respondents (80.0%) agreed that modern biotechnology should be used to improve crops in Bangladesh. 5.1% did not think innovation through modern biotechnology is needed, while 14.9% did not pose any comments. Those who support the technology think that rice, potatoes, and tomatoes should be improved through modern biotechnology. 55.4% respondents opined biotechnology should be the method to get rid of eggplant insects. The respondents also felt the necessity of guidelines. 82.7% of respondents opined that there should be guidelines for Bt Brinjal cultivation, storage, marketing, and modern biotechnology research. 2.9% said the guidelines are not required, and 14.4% of respondents did not provide any opinion.

## Discussion

Modern biotechnology demonstrates great potential in numerous fields, providing inventive solutions to urgent issues in agriculture, healthcare, sustainable livelihoods, and industrial applications (Nawaz et al., 2022). GE crops were first commercialized in the mid-1990s and tested or adopted in more than 30 countries, with various benefits (Smyth, Kerr, and Phillips, 2015). Society cannot realize the potential of technology without consumer acceptance. Thus, understanding the factors that contribute to customers’ reluctance is crucial for developing GE products in the future (Verdurme and Viaene, 2003). While a negative attitude toward GE crops is perpetuated, it is often associated with insufficient knowledge of modern technologies, a lack of trust in regulators, inadequate communication regarding the risks and/or benefits, and personal values. The cumulation of these factors can seriously impact food security issues (Shohael and Hefferon, 2023).

With around 170 million people living in a land area of 148,460 square kilometers, Bangladesh is the eighth most populous country globally and one of the most densely populated (Wikipedia contributors, 2024). Being a predominantly agricultural nation, Bangladesh’s economy depends on agricultural production, which generates 19.6% of the country’s GDP and employs 63% of its labor force. The introduction of Bt Brinjal in Bangladesh marked a significant event as agricultural biotechnology implications moved forward. Bt Brinjal varieties have been cultivated since 2014 in Bangladesh; so far, no evidence of any unaccepted or undesirable effects that might harm human health, animals, or the environment has been reported. Farmers cultivating Bt Brinjal are pleased with the performance and profit (M. R. I. Mondal and Nasrin, 2018). Bangladesh’s deployment of GE crops to boost agricultural productivity and less pesticide use could serve as a model for other developing nations facing similar challenges (Ahmed et al., 2019). However, the public should be sensitized and informed with science and evidence-based information to proceed further.

Most people are unaware of the frequency of insecticide spraying during Brinjal cultivation. It is common practice in Bangladesh for conventional Brinjal crops to be sprayed with insecticides more than 80 times during the 4–5-month growing season in all the main cultivation regions (Meherunnahar and Paul, 2009). Farmers have noted that growing Bt Brinjal has led to better insect control, lower labor and chemical expenses, higher yields, and increased income. They are pleased with the quality of Brinjal they produce, which they can offer at a lower price. With fewer pesticides needed, farmers feel that Bt-Brinjal is safer for human health (Haque and Saha, 2020). This information should be appropriately communicated to the public so that they can realize the actual benefits that Bt Brinjal aims to provide. The Government of Bangladesh has demonstrated a willingness to adopt and implement modern agricultural policy frameworks and guidelines. The country is mandated to support the safe and appropriate use of science and technology, including modern biotechnology, to help meet agricultural challenges, as implicated by the National Agricultural Policy (2018).

Before discussing the findings, it should be noted that the demographics of the present study are skewed by highly educated individuals because the surveys were conducted primarily at the university and nearby areas, and this does not represent the general population. However, the present study observed some interesting facts and beliefs among this group of respondents. The present study showed that most of the people who knew about the crop were students (higher secondary or above), and a majority of them had a science background. This is because the curricula contain chapters regarding biotechnology from secondary schools, with information about GE crops and Bt Brinjal. Moreover, efforts included information campaigns conducted through various channels, including mass media, radio, television, and printed materials such as pamphlets and posters. It was evident from the study that the familiarity of the Bt Brinjal was mostly from coursework or mass media. In addition, this implies that laypeople who don’t have access to the curriculum or promotional materials are not familiar with it. Therefore, more innovative measures such as combining government support, extension services, demonstration plots, information campaigns, success stories, and research efforts may increase people’s familiarity with Bt Brinjal in Bangladesh. Though familiarity is demonstrated, the idea or proper knowledge of the technology is not accurate. Therefore, disseminating the science behind the technology may not have been appropriately addressed. The success of any technology requires proper communication among laypeople.

Though the success of Bt Brinjal has been demonstrated in many previous studies (M. R. I. Mondal and Nasrin, 2018; Ahmed et al., 2019; Shelton et al., 2020), a large portion of the consumers in the present study could not confirm that they ever consumed Bt Brinjal. There are practical challenges in labeling the product, as Brinjal is a highly consumed and cheap vegetable sold in bulk in every corner of the country. Therefore, alternative measures may help create a positive appeal so that people can buy and eat without hesitation and make an informed choice.

The interview revealed that those familiar with the crop were also aware of the absence of health risks and nutritional differences. Research indicates that significant portions of consumers lack awareness or a clear understanding of GMOs and their traits and effects (Ribeiro, Barone, and Behrens, 2016; Hwang and Nam, 2021). People express their favorable impression of the technology as they expect the Bt Brinjal to be increasingly popular in the market and may influence other non-GE varieties. However, many respondents did not provide any insights in response to the questions regarding the popularity and influence of Bt Brinjal in the market. This implies a lack of confidence in giving any opinion, as they had no concrete knowledge about the matter. This uncertainty can stem from various factors, such as limited access to reliable information, conflicting sources, or complexity of the subject matter. As a result, individuals may refrain from engaging in discussions or taking positions until they have acquired sufficient knowledge and understanding to form informed opinions. The lack of confidence was also evident when people’s preference for buying skewed to local/native Brinjal varieties. It was also apparent that people with previous scientific knowledge were more inclined to buy Bt Brinjal. Therefore, continuous counseling may help increase the confidence of consumers.

Many consumers also express dissatisfaction with their own knowledge on the subject, highlighting a need for broader consumer education efforts (Wunderlich and Gatto, 2015). Therefore, creating awareness about any GE crops is critical, as it provides balanced information in accessible language through various channels, emphasizes scientific consensus, encourages critical thinking, and respectfully addresses concerns. As the current study revealed people were eager to receive information and embrace good science, the source of information should be accurate and authentic. Negative perceptions significantly impact how GE foods are viewed (Giordano et al., 2018). These perceptions are resistant to change, even when consumers are presented with new information (Grunert, Bredahl, and Scholderer, 2003). Moreover, the scarcity of information about GE products partly stems from scientific uncertainty caused by conflicting sources of information (Palmieri et al., 2020).

These interviews demonstrated positivity toward modern biotechnology. This is supported by their idea of different problems associated with different types of crop cultivation in Bangladesh. They think technological interventions could solve the problems. This implies that a supportive stance toward biotechnology and GM crops may help realize the potential benefits and address pressing issues.

## Conclusion

The study highlights a gap in public knowledge and awareness about Bt Brinjal and modern biotechnology. Individuals with science backgrounds have a better understanding and appreciation of biotechnology. Therefore, enhancing communication with scientific evidence and improving science education can address misconceptions and improve community perception. To leverage biotechnology for a sustainable agricultural sector that meets Bangladesh’s growing population needs, it is essential to educate the public, enabling them to make informed decisions.

## Data Availability

The raw data supporting the conclusions of this article will be made available by the authors, without undue reservation.
